# Relación entre conducta alimentaria y potencial cariogénico en la primera infancia. Una revisión

**DOI:** 10.21142/2523-2754-1304-2025-267

**Published:** 2025-11-08

**Authors:** Ninoshka Darling Guanoluisa-Barragán, Pamela Lisbeth Albán-Zambrano, Francisco Tinajero-Moscoso, Cecilia Molina-Jaramillo, Darwin Luna-Chonata

**Affiliations:** 1 Universidad Hemisferios. www.fjosue2012@hotmail.com dvlunac@uhemisferios.edu.ec ninoshk_99g@hotmail.com Universidad de Los Hemisferios Universidad Hemisferios Ecuador www.fjosue2012@hotmail.com dvlunac@uhemisferios.edu.ec ninoshk_99g@hotmail.com; 2 Universidad Regional Autónoma de los Andes. pamealban21@gmail.com Universidad Regional Autónoma de Los Andes Universidad Regional Autónoma de los Andes Ecuador pamealban21@gmail.com; 3 Universidad Central del Ecuador. cbmolinaj@uce.edu.ec Universidad Central del Ecuador Universidad Central del Ecuador Ecuador cbmolinaj@uce.edu.ec

**Keywords:** Caries Dental, Nutrición Infantil, Salud del Niño, Hábitos Alimentarios, Dental Caries, Infant Nutrition, Child Health, Feeding Behavior

## Abstract

**Introducción:**

La conducta alimentaria engloba comportamientos individuales y grupales asociados al uso y consumo de alimentos. El análisis de estos patrones alimentarios constituye una herramienta fundamental para obtener información precisa y relevante que permita evaluar el estado dental y nutricional de un grupo de individuos. El objetivo de este estudio fue analizar la relación existente entre la conducta alimentaria y su potencial cariogénico en la primera infancia, mediante una revisión narrativa de la literatura científica publicada entre 2017 y 2024.

**Materiales y Métodos:**

Se consultaron las bases de datos Medline, LILACS y ScienceDirect, utilizando términos de búsqueda combinados mediante operadores booleanos y filtrando los resultados por metaanálisis, revisiones sistemáticas, estudios observacionales, estudios de casos, ensayos clínicos aleatorizados y estudios transversales.

**Resultados:**

De los 106 artículos obtenidos, se seleccionaron 9 que cumplían con los criterios de inclusión y exclusión establecidos. Los resultados de esta revisión indican la necesidad de promover una alimentación adecuada desde las primeras etapas de vida para prevenir enfermedades dentales en la infancia. Asimismo, se destaca la importancia de fomentar los cuidados de salud oral, incluyendo técnicas de cepillado correctas, tanto en padres como en cuidadores.

**Conclusiones:**

La educación en salud oral es un pilar fundamental para abordar este importante problema de salud pública.

## Abstract (en)

**Introduction:**

Feeding behavior encompasses a set of individual and group actions related to food use and consumption. Analyzing these dietary patterns serves a crucial tool for obtaining precise and relevant data to assess the dental and nutritional status of a population. We aimed to examine the relationship between dietary behaviors and their cariogenic potential in early childhood through a narrative review of scientific literature published among 2017 and 2024.

**Materials and Methods:**

Medline, LILACS, and ScienceDirect databases were searched using Boolean operators to combine search terms and filter results by meta-analyses, systematic reviews, observational studies, case studies, randomized controlled trials, and cross-sectional studies. Of the 106 articles retrieved, we included 9, because those met the inclusion and exclusion criteria.

**Results:**

The results highlighted the need to promote proper nutrition from early life to prevent dental diseases in children. Additionally, we emphasize the importance of fostering oral health care, including correct brushing techniques, among both parents and caregivers.

**Conclusion:**

We concluded that oral health education is a cornerstone for addressing this significant public health issue.

## INTRODUCCIÓN

La conducta alimentaria se refleja en comportamientos individuales y grupales relacionados con el uso y consumo de alimentos, los cuales forman parte de las prácticas socioculturales ^1^. Estos patrones han experimentado modificaciones debido a distintas variables que inciden en la dinámica y la convivencia ^2^. Uno de estos factores es la situación económica que influye en los patrones de consumo tanto en niños como en adultos ^3^. Otra variable es el tiempo de dedicación y tiempo invertido por los padres, relacionados con el consumo de alimentos poco saludables ^4^.

La alimentación es una necesidad básica para todos los seres humanos y representa un factor clave que determina el estado nutricional de una persona ^5^. En este sentido, el análisis del consumo alimenticio se presenta como una herramienta valiosa para obtener información confiable y oportuna, esencial para evaluar la condición dental y nutricional en un grupo de individuos ^6^.

De esta manera, una alimentación saludable no solo resulta fundamental para mantener un buen estado de salud en general, sino también para preservar la salud oral ^7^. Una dieta adecuada o inadecuada se relaciona con la probabilidad de desarrollar enfermedades bucales, como caries dentales o afecciones de las encías, en cualquier etapa de la vida ^8^. La relación entre la dieta y la cultura alimentaria durante la infancia temprana se vincula con estos problemas de salud oral, así como con hipoplasia del esmalte, disfunción de las glándulas salivales y retraso en la erupción dental, incluso se ha observado que deficiencias nutricionales de proteínas y alimentos energéticos, inciden en el desarrollo de patologías en la cavidad bucal ^9^, siendo también importante la dieta durante el estado de gestación para garantizar el desarrollo dental normal del producto en formación ^10^.

En este contexto, es esencial que durante su crecimiento y desarrollo el niño reciba una cantidad y calidad adecuada de nutrientes, lo que se relaciona con un sistema capaz de recibir, absorber, metabolizar y excretar los componentes presentes en la dieta, siendo la nutrición la capacidad de adaptación del ser humano al medio ambiente ^11^. De esto parte la cultura alimentaria poco saludable que se vincula directamente como factor de riesgo para diversas enfermedades crónicas, las cuales también repercuten en la salud bucal ^12^, como el desarrollo de caries dental en niños vinculado con el consumo de bebidas con alto contenido de azúcar durante el primer año de vida y con un riesgo que persiste a lo largo de la vida ^11^. En este sentido, el potencial cariogénico está en relación directa con la interacción entre cultura alimentaria, calidad de nutrientes en la dieta, falta de higiene bucal adecuada y acumulación de biofilm dental ^13^.

En ese sentido, el cuidado dental incide significativamente en el crecimiento y desarrollo cognitivo a lo largo del tiempo en los niños, interfiriendo con su nutrición, masa corporal y crecimiento en estatura ^14^. La desnutrición y la malnutrición infantil tienen un pronóstico reservado; la malnutrición tiene un concepto más amplio y ocurre cuando las personas tienen una alimentación inadecuada en la que faltan o sobran nutrientes provocando efectos negativos en la salud y el desarrollo, mientras que la desnutrición como una forma de malnutrición, se produce por deficiencias de nutrientes y/o micronutrientes, y afecta gravemente a la supervivencia y desarrollo infantil ^15^. 

Frente a ello, el objetivo del presente estudio fue analizar la correlación entre cultura alimentaria y el potencial cariogénico en la primera infancia mediante una revisión narrativa. 

## MATERIALES Y MÉTODOS

**Figura 1:**
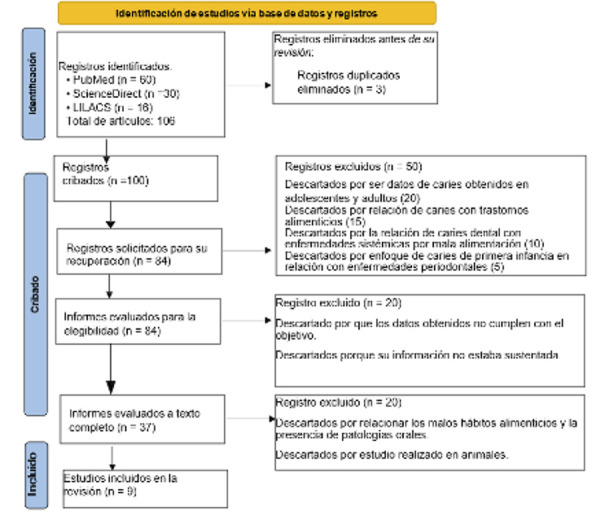
Diagrama de flujo PRISMA 2020; muestra el proceso de búsqueda, selección y exclusión de los artículos utilizados en la revisión narrativa. Se especifican las bases de datos consultadas, el número total de estudios recuperados, los criterios de exclusión aplicados y los estudios finalmente incluidos.

Se realizó una revisión narrativa de la literatura sobre desnutrición y malnutrición y su relación con la caries dental, buscando analizar las manifestaciones orales relacionadas con la deficiencia alimentaria, abordando tanto la salud oral como sistémica. Los criterios de inclusión abarcaron artículos publicados entre los años 2017 y 2024 en español e inglés sobre cultura alimentaria y patologías orales en la primera infancia; le tipo metaanálisis, revisiones sistemáticas, estudios observacionales, estudios de casos, ensayos clínicos aleatorizados y estudios transversales. Se excluyeron estudios en otros idiomas o de otros tipos como: investigaciones sobre población adolescente, adulta o adulta mayor, así como aquellos sin seguimiento en los individuos o realizados en animales. La búsqueda se realizó en Medline, ScienceDirect y LILACS, siguiendo como referencia el diagrama PRISMA 2020 (Figura 1).

*Este estudio no requirió aprobación por comité de ética institucional, ya que se basa exclusivamente en el análisis de artículos previamente publicados*.

Para desarrollar la búsqueda, se vinculó palabras críticas del tema de investigación, como “feeding habits” y “dental caries”, en inglés y español, articulados con operadores Booleanos “AND”/“OR” y filtrando según los tipos de publicaciones, como se detalla en la Tabla 1.

**Tabla 1:** Parámetros de Metodología PRISMA 2020; se detallan los criterios de elegibilidad, las fuentes de información, cadenas de búsqueda utilizadas en cada base de datos, proceso de selección y recolección de datos, así como los términos MeSH empleados.

Sección	Ítem
Criterios de elegibilidad	Enfocado en los criterios de inclusión y exclusión.
Fuentes de Información	Medline, ScienceDirect y LILACS
Cadena de Búsqueda	Medline:
	(Odontology OR
	temporary dentition) AND
	(Feeding Habit OR, Pediatric dentistry)
	AND (Cavities).
	ScienceDirect:
	(Feeding Habits) AND (temporary dentition)
	(Feeding Habits) AND
	(dental caries)
	LILACS:
	(nutritional condition) AND (temporary dentition) OR
	(salud bucal) AND
	(caries de la primera infancia)
Proceso de selección	Se eliminaron los artículos duplicados y aquellos que no corresponden con el tema de investigación.
	Se realizó un análisis de títulos y resúmenes.
	La información relevante de los artículos seleccionados se recopiló mediante una revisión exhaustiva de cada uno de ellos.
Proceso de recolección de datos	Mediante una minuciosa organización en un estado del arte en Microsoft Excel.
Términos MeSH	Cariogenic potential /cavities, / Feeding Habits/ poor nutrition/ malnutrition / temporary dentition/ Odontology / pediatric dentistry.
Métodos de síntesis	Los resultados de los estudios utilizados se organizaron en tablas para facilitar una comprensión más clara y efectiva.

## RESULTADOS

Se obtuvieron un total de 106 artículos, mediante el uso de las cadenas de búsqueda definidas en la Tabla 1, siendo 60 de Medline, 30 de ScienceDirect y 16 de LILACS. 

Tomando como referencia a la metodología PRISMA 2020, se sometió a los artículos a un proceso de evaluación y elección, verificando fundamentalmente los criterios de inclusión y exclusión anteriormente mencionados, de acuerdo con lo plasmado en la Figura 1

Finalmente se incluyeron 9 artículos pertinentes al estudio, asegurando que cumplan con los criterios de inclusión, siendo la mayoría de tipo transversal, como se detalla en Tabla 2 los datos obtenidos de cada uno.

**Tabla 2:** Resultados de los artículos incluidos.

Nº	Autor	Año de publicación	Características de la población	Diseño del estudio	Metodología	Resultados	Conclusiones
1	Giacosa K, et al (16)	2024	50 infantes de 1 año a 2 años , de sexo masculino y femenino	Estudio transversal	Se analizó el índice de caries mediante los criterios de ICDAS, índice placa visible e índice PUFA, registrados en digital.	Los niños que consumían alimentos sólidos con azúcar presentaron, en promedio, dos dientes más afectados en comparación con aquellos que los consumían con más frecuencia.	Cuanto más a menudo se consumen bebidas y alimentos azucarados, mayor es la probabilidad de tener caries y lesiones más graves.
2	Velez L, et al (17)	2023	600 niños preescolares urbanas y rurales	Estudio observacional y transversal	Se evaluó mediante datos recogidos al realizarse una encuesta epidemiológica en el 2019 a estudiantes del sur del Ecuador.	Se descubrió que la tasa promedio de prevalencia de caries en niños preescolares es del 23,8% en menores de 3 años, y del 57,3% en aquellos de 3 a 6 años.	La desnutrición es una preocupación de salud significativa, lo que ha llevado a altos niveles de prevalencia y experiencia de CIT.
3	de Oliveira, et al (18)	2023	533 niños en etapa pre-escolar (de 4 a 6 a.)	Estudio transversal	Se evaluó la relación de una alimentación en los primeros 6 meses.	El estudio demostró que el infante que se alimentaba con azúcares antes de los 6 meses de edad tenía un 58% de presentar CIT, en comparación con aquellos que no lo fueron.	La alimentación complementaria con azúcares antes de los 6 meses genera con mayor susceptibilidad de CIT.
4	Wang, et al (19)	2022	Infantes de 2-5 años. Con un total de 150.	Estudio transversal	Se evaluó la ingesta nutricional de cada niño por 24 h, y se los dividió por 3 grupos: caries, grupo con CIT, y el de caries severa.	Se evidenció que CIT se relacionaron con un notable desequilibrio en la dieta y un consumo elevado de cereales, así como una mala nutrición.	La muestra de estudio experimenta un problema significativo de salud relacionado con mal equilibrio en su alimentación.
5	Zou J, et al. (20)	2022	193 datos proporcionados por las Naciones Unidas desde el 2007 y 2017.	Estudio transversal	Revisión sistemática de la prevalencia de CIT, basados en diferentes evaluadores de caries como: CAT, CAMBRA, ADA y cariograma.	La elección de alimentos tiene un fuerte vínculo con la Caries Dental de la Primera Infancia y consumir una dieta equilibrada que incluya proteínas magras y verduras beneficia la salud oral.	El consumo frecuente de una mala nutrición con azucares y jugos, aumentó el riesgo de desarrollar CIT.
6	Arévalo P et al (21)	2021	Estudios de Infantes desde los 0-3 años.	Revisión de literatura	Se evaluó el hábito nutricional del efecto del consumo de azúcares y su relación consecuente de CIT mediante datos bibliográficos.	La caries de infancia temprana comienza poco después de la erupción dental y se transmite con mayor frecuencia verticalmente, siendo el microorganismo más cariogénico el Streptococcus mutans.	Se demostró que la desnutrición no se relaciona directamente con la caries de primera infancia. Sin embargo, el riesgo de caries se asocia con el consumo excesivo de azúcares.
7	Robalino-Tello et al (22)	2021	393 bases científicas	Revisión de literatura	Se evaluaron los resultados obtenidos de revisiones bibliográficas del 2017 al 2021.	La mala nutrición, está relacionada con la caries de la primera infancia causada por alimentos con alto contenido de azúcar en la dieta.	El excesivo consumo de sacarosa tiene un impacto significativo en el desarrollo de CIT.
8	Wulaerhan J, et al (23)	2020	Infantes de 3-5 años, con un total de 670.	Estudio Transversal	Mediante un examen completo y con el uso del índice “ceod” se evaluó la caries dental y se diagnosticó las CIT, con los criterios establecidos por la Academia Estadounidense de Odontología Pediátrica.	Se encontró una relación importante entre la Caries Dental de la Primera Infancia y la frecuencia de una mal nutrición que incluya: golosinas, chocolate, aguas endulzadas, y exceso de lácteos.	La alta frecuencia de consumo de frutas, agua azucarada y leche, combinada con prácticas déficit de higiene y la ausencia de medidas preventivas, influye en el deterioro de la salud bucal en este grupo de personas.
9	Tsang, Chloe et al. (24)	2019	Infantes de 6 meses a 6 años. Con un total de 836 niños.	Estudio transversal	Se realizó una evaluación de la salud bucal y relación con la nutrición de niños pequeños.	Los análisis dentales mostraron que entre el 52% y el 79% de los infantes de 5 a 6 años presentaban CIT. Se identificaron vínculos entre CIT y desnutrición en niños. La desnutrición en niños menores de 5 años se manifiesta con un 41% de retraso en el crecimiento, un 29% de bajo peso y un 11% de emaciación, destacando su prevalencia en zonas rurales.	Se encontraron índices elevados de desnutrición y presencia común CIT en niños de 5 a 6 años, particularmente en zonas rurales.

CIT: “Caries de la Infancia Temprana”. ICDAS: Sistema Internacional de Detección y Evaluación de Caries. PUFA: Exposición pulpar (P), ulceración de la mucosa oral por fragmentos radiculares (U), Fístula (F), Absceso (A). CAT: Herramienta de evaluación de caries. CAMBRIA: Manejo de caries por evaluación de riesgo. ADA: Asociación Dental Americana.

De manera general, coinciden en la fuerte asociación entre el consumo de azúcar ^16^ y el desarrollo de caries dental en la primera infancia, subrayando el papel crucial de la dieta en el desarrollo de esta enfermedad ^17^, independientemente del tipo de estudio. Sobre todo, el consumo de azúcares entre comidas ^18^, así como la frecuencia y duración de la exposición a azúcares fermentables ^19^ ya que favorecen la proliferación de bacterias cariogénicas en boca ^(16, 20)^ incrementando el riesgo de desmineralización del esmalte dental ^21^.

Además de la dieta, otros factores como la higiene bucal inadecuada y falta de acceso a servicios de salud oral, también influyen en la prevalencia de caries dental entre la población ^22^. La desnutrición, aunque no se relaciona directamente con la caries dental, puede coexistir con ella ^23^, sobre todo en poblaciones vulnerables ^24^. En general, los estudios resaltan la importancia de promover una alimentación saludable desde las primeras etapas de la vida, con un bajo contenido de azúcares añadidos; así como fomentar hábitos de higiene oral adecuados para prevenir caries en la temprana infancia.

## DISCUSIÓN

La caries de infancia temprana (CIT) continúa siendo un problema relevante de salud pública, cuya aparición se relaciona con la erupción dental temprana y la transmisión vertical del *Streptococcus mutans*, tal como lo demuestra Giacosa et al. en su estudio piloto ^16^. Este hallazgo fue confirmado en esta revisión, donde se identificó que los niños expuestos a prácticas alimentarias cariogénicas presentaron mayor prevalencia de CIT.

Estos hallazgos destacan que el consumo excesivo de azúcares está fuertemente relacionado con la aparición de caries en la infancia, lo cual coincide con lo reportado por Vélez et al. ^17^ y de Oliveira et al. ^18^, quienes observaron una mayor incidencia de caries en niños con introducción temprana de azúcares. Esto valida la relación entre hábitos dietéticos inadecuados y riesgo cariogénico en la primera infancia.

Sin embargo, Arévalo et al. ^21^ señalan que la desnutrición por sí sola no se relaciona directamente con la aparición de caries, lo que contrasta con lo evidenciado por Wulaerhan et al. ^23^, quienes encontraron una asociación significativa entre malnutrición y CIT, especialmente en contextos donde la dieta es rica en carbohidratos fermentables. Esta discrepancia se presenta por las diferencias contextuales y metodológicas de los estudios incluidos, así como por el papel mediador de otros factores como la higiene oral y el acceso a servicios de salud.

Asimismo, este estudio corrobora lo planteado por Zou et al. ^20^, quienes argumentan que una dieta equilibrada rica en vegetales y proteínas reduce el riesgo de CIT. Esta evidencia refuerza el valor protector de patrones dietéticos saludables como la dieta mediterránea, cuyos efectos beneficiosos también fueron destacados por Vélez et al. ^17^.

En cuanto a la influencia de factores socioculturales y económicos, nuestros hallazgos coinciden con lo reportado por Tsang et al. ^24^, quienes evidenciaron que en zonas rurales la combinación de pobreza, baja escolaridad de los padres y limitada cobertura de servicios de salud bucal incrementa la prevalencia de caries. No obstante, también se evidenció que intervenciones comunitarias y educativas pueden reducir estos efectos, lo cual se alinea con lo propuesto por Puruncajas-Armas et al. ^25^.

Por otro lado, aunque la mayoría de los estudios incluidos no reportan diferencias significativas entre sexos, algunos como el de Vélez et al. ^17^ identifican una ligera mayor prevalencia en varones, mientras que Wulaerhan et al. ^23^ no hallaron asociación significativa. Estas inconsistencias sugieren que el sexo, por sí solo, no constituye un factor determinante, aunque podría estar mediado por variables conductuales o ambientales.

Finalmente, esta revisión reafirma la necesidad de iniciar la prevención de caries desde etapas prenatales ^25^, tal como lo promueve la literatura actual ^26^, mediante estrategias de educación nutricional y promoción de la salud oral, tanto en el hogar como en instituciones educativas ^27^. Aunque el número de artículos incluidos es limitado, la aplicación rigurosa de los criterios PRISMA aporta solidez metodológica a los hallazgos.

## CONCLUSIÓN:

El presente estudio reveló la correlación existente entre la cultura alimentaria y la caries de la primera infancia, esclareciendo que no solo el consumo excesivo de alimentos de manera desequilibrada, sino que también la falta de nutrientes, e inadecuada alimentación en el infante, causa caries de la primera infancia y puede generar una abrupta irrupción en su crecimiento y desarrollo a largo y corto plazo, mismos que pueden ser irreversibles en el desarrollo cognitivo e intelectual.

## Financiamiento:

Autofinanciado
